# Nonlinear model predictive control with logic constraints for COVID-19 management

**DOI:** 10.1007/s11071-020-05980-1

**Published:** 2020-12-02

**Authors:** Tamás Péni, Balázs Csutak, Gábor Szederkényi, Gergely Röst

**Affiliations:** 1 Institute for Computer Science and Control (SZTAKI), Kende u. 13-17, Budapest, 1111 Hungary; 2 Department of Control for Transportation and Vehicle Systems, Faculty of Transportation Engineering and Vehicle Engineering, Budapest University of Technology and Economics, Stoczek u. 2, Budapest, 1111 Hungary; 3 Faculty of Information Technology and Bionics, Pázmány Péter Catholic University, Práter u. 50/a, Budapest, 1083 Hungary; 4 Bolyai Institute, University of Szeged, Szeged, 6720 Hungary

**Keywords:** COVID-19, Epidemic model, Disease control, Differential equations, Control theory, Model predictive control, Temporal logic

## Abstract

The management of COVID-19 appears to be a long-term challenge, even in countries that have managed to suppress the epidemic after their initial outbreak. In this paper, we propose a model predictive approach for the constrained control of a nonlinear compartmental model that captures the key dynamical properties of COVID-19. The control design uses the discrete-time version of the epidemic model, and it is able to handle complex, possibly time-dependent constraints, logical relations between model variables and multiple predefined discrete levels of interventions. A state observer is also constructed for the computation of non-measured variables from the number of hospitalized patients. Five control scenarios with different cost functions and constraints are studied through numerical simulations, including an output feedback configuration with uncertain parameters. It is visible from the results that, depending on the cost function associated with different policy aims, the obtained controls correspond to mitigation and suppression strategies, and the constructed control inputs are similar to real-life government responses. The results also clearly show the key importance of early intervention, the continuous tracking of the susceptible population and that of future work in determining the true costs of restrictive control measures and their quantitative effects.

## Introduction

On December 31, 2019, China alerted the World Health Organization (WHO) on a cluster of pneumonia cases of unknown origin in Wuhan, China. On January 7, 2020, the causative pathogen of the outbreak was identified as a novel coronavirus, later named as SARS-CoV-2, and the disease it causes as COVID-19. SARS-CoV-2 infections quickly spread: the first case outside China was identified in Thailand, on 14 January, followed by reported cases from a number of countries [6, 56].

In Europe, the first cases were confirmed on January 24, 2020, in France (where, later in April, COVID-19 was retrospectively confirmed for a patient hospitalized in late December 2019) [13, 50], and on January 27, in Germany, Bavaria, leading to a local outbreak [7]. The first epidemic in Europe started in the Lombardy region of Italy with the first detection on February 20, 2020 [46]. Control measures started in mid-March in most of the European countries, including social distancing measures that reflect strong effort to suppress, or at least to slow down the spreading of COVID-19. Because of the differences in timing and stringency of the applied measures, the peak daily incidence varied substantially among countries, and recently a resurgence of cases has been observed [17]. By the end of July 2020, around seventeen million cases and seven hundred thousand deaths have been reported worldwide, with significant spreading in the Americas, Eastern Mediterranean and Southeast Asia [57].

In the absence of vaccine and effective treatment, the non-pharmaceutical intervention strategies can roughly be divided into two main categories. Mitigation does not aim to completely stop the transmission of the virus, only to slow down to keep the number of infected people below the capacity of the healthcare system. Sweden is an example of such strategy. On the other hand, suppression aims to reduce the incidence to a very low level by strict social distancing and then keep that number low by localized and targeted measures, such as efficient surveillance, testing, tracing and quick isolation of cases. The first outbreak was suppressed in most European and East Asian countries, Australia and New Zealand. Recently, following a relaxation of such measures, a resurgence has been observed in the Western Balkans [17].

Mathematical models have been commonly used in epidemiology to evaluate disease control strategies. However, disease control in this context usually refers to a single intervention measure that is sufficient to reduce the reproduction number below one, leading to the eradication of the disease. The most commonly used measures are vaccination and drug treatment [19], or, in the case of vector borne diseases, culling of mosquitoes and other arthropods that transmit the pathogen into other living organisms. The current COVID-19 situation is unprecedented in the sense that governments are constantly tuning their control measures, trying to find balance between public health concerns and the costs of social distancing measures to the society and the economy. Thus, using feedback, which is a standard tool in control theory, is necessary to dynamically manage our response to the pandemic and tailor policies to stabilize the situation.

In a control theory framework, dynamical systems are considered as operators mapping from an input signal (function) space to an output space [48]. We distinguish between manipulable inputs which can be set (often between certain limits) by the user and disturbance inputs from the environment that cannot be directly influenced. The outputs are either directly measured quantities or they are computed from measurements. The control goals are usually prescribed using the outputs, e.g., they have to track a reference trajectory or just stay between predefined limits. Such goals are often equipped with additional constraints and optimality criteria. Possible examples for the former are (physical) bounds on the inputs and/or on the state variables, and minimal control cost or operation time for the latter. Therefore, a complex control problem can be most often expressed in the form of constrained optimization.

Even the simplest epidemic models are nonlinear which makes the corresponding control problems challenging due to complex dynamical behavior, possible singularities and the state-dependent nature of fundamental properties like reachability or observability [30]. Parameter and input uncertainties, or the lack of measurements of sufficient quality often add further difficulties to the problem [42, 44].

There is a wide literature on the model-based targeted manipulation of diseases either within the host or across an entire population [1, 4, 27, 41, 49, 53]. Nonlinear model predictive control (NMPC) is introduced in [47] as a potential tool for epidemic management. In [8] NMPC is used for the optimal allocation of vaccination resources between different risk groups and regions. A robust model predictive approach for stochastic epidemic models is proposed in [54], where quarantine policy design is shown as a possible control input. Detailed control-related model analysis and vaccination input design are proposed in [12] which tracks a prescribed output given in terms of susceptible and infected people. A quantitative model is presented in [52] for the COVID-19 outbreak in Wuhan, China, taking into consideration the effect of different interventions. In [22] an eight-compartment ODE model is presented for describing and analyzing the COVID-19 epidemic in Italy, where the authors show different scenarios for the implementation of countermeasures. The same model structure is used in [32] adapted to the data from Germany. A model predictive control approach is proposed, and it is shown that the number of fatalities can be significantly reduced even when the model and some measurements are uncertain. Vast majority of the available control approaches assume a control input with continuous range which is clearly useful for strategic planning, but not straightforward to put into practice if there are distinct levels of intervention. A notable exception is [37], where starting and stopping strict social distancing is a binary control input applied in a nonlinear model predictive framework and tested through simulations on nominal and uncertain models of the COVID-19 pandemic in Brazil.

Most advanced feedback control methods need the whole state information for computing the input, but it is not realistic to assume that the number of individuals in each compartment can be continuously measured (especially latent, asymptomatic or even mildly symptomatic people). Therefore, a state estimator is needed in practice, which is known to be non-trivial to design for nonlinear systems, and most often its stability has to be proved on a case by case basis [30]. A general observer class with convergence proof is proposed for low-dimensional continuous time epidemic models in [29]. An implicit observer design approach for specially discretized SEIR models with global convergence proof is described in [28].

Temporal logic provides a powerful framework for the modeling, analysis and control of discrete time dynamical systems, which is a correct-by-construction approach [5]. Using signal temporal logic, complex specifications and constraints can be given for the required dynamical behavior of a model in a compressed form. A particularly successful application of this computation framework is model predictive control, where the requirements can be automatically translated to a mixed integer programming problem taking into consideration the system dynamics as constraints [18]. Most often, linear dynamical models are preferred for control design with temporal logic, since those can be put into the framework of mixed integer linear programming. However, there exist really powerful solvers capable of efficiently handle nonlinear models as well [33].

Based on the above, the aim of this paper is to propose an optimization-based control approach for compartmental epidemic models constructed for the COVID-19 outbreak, which is able to take into account complex, possibly time-dependent specifications including bounds, and even logical relations between model variables, and multiple predefined discrete levels of interventions. Another important goal is to study the possibilities of output feedback design by applying a dynamic state observer. As a case study, we parameterize our model to Hungary, but it can be easily generalized to other countries as well.

**Fig. 1:**
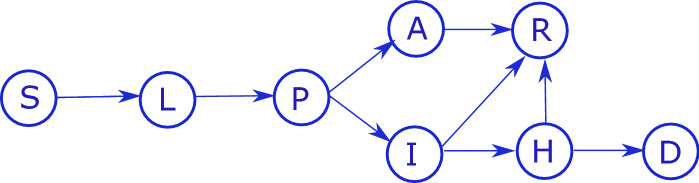
Transition diagram. Circles represent compartments, and arrows represent transitions between these compartments

## Transmission dynamics model

### Model description

We construct a compartmental model to describe the transmission dynamics of the infection, incorporating specific characteristics of COVID-19. Our population *N* is divided into the following classes, tracking the disease status of individuals: by *S* we denote the susceptibles, i.e., those who can be infected by the disease. Latent (*L*) are those who have already contracted the disease but do not show symptoms and are not infectious yet. Since transmission may occur in the two days before the onset of symptoms [2], we consider a pre-symptomatic infectious compartment *P*. Since a large fraction of infected show only mild or no symptoms, after the incubation period, we differentiate infected individuals into asymptomatic (*A*) and symptomatic infected (*I*) compartments. Those in *A* will always recover, while the more severe cases in *I* may require hospitalization, in which case they move to compartment *H*, from where they may eventually recover (*R*) or die (*D*). We note that most transmission occurs within a few days after symptom onset, and the compartment *I* reflects this period of effective infectivity, rather than clinical status or PCR positivity, which may continue for weeks, yet we remove them from *I* and place them in *R* as they do not participate in chains of transmission anymore. The transition diagram of our model is depicted in Fig. 1. Several studies [3, 14, 39, 40, 45, 55] have proposed somewhat similar models for of COVID-19.

The compartmental model without any control terms reads as

1 $$\begin{aligned} S'(t)= & {} - \beta \left[ P(t) + I (t) + \delta A(t) \right] S(t)/N, \end{aligned}$$

2 $$\begin{aligned} L'(t)= & {} \beta \left[ P(t) + I (t) + \delta A(t) \right] S(t)/N- \alpha L(t), \nonumber \\ \end{aligned}$$

3 $$\begin{aligned} P'(t)= & {} \alpha L(t) - p P(t), \end{aligned}$$

4 $$\begin{aligned} I'(t)= & {} q p P(t) - \rho _I I(t), \end{aligned}$$

5 $$\begin{aligned} A'(t)= & {} (1-q) p P(t) - \rho _A A(t), \end{aligned}$$

6 $$\begin{aligned} H'(t)= & {} \rho _I \eta I(t) -h H(t), \end{aligned}$$

7 $$\begin{aligned} R'(t)= & {} \rho _I (1-\eta ) I(t) + \rho _A A(t)+ (1-\mu )h H(t),\nonumber \\ \end{aligned}$$

8 $$\begin{aligned} D'(t)= & {} \mu h H(t). \end{aligned}$$

### Model parameters

From the infectivity profile of COVID-19 [2], we can see that most transmissions occur between 3 days prior to and 4 days after symptom onset, with the pre-symptomatic infection fraction 43.7%. It is a good approximation to set the pre-symptomatic period $p^{-1}$ as three days, and the symptomatic infectious period $\rho _I^{-1}$ as four days, with the same infectiousness $\beta$ during this period. The estimated mean incubation period (which is the latent and pre-symptomatic period together) of the coronavirus disease is 5.5 days [35]; thus, the latent period $\alpha ^{-1}$ is 2.5 days. Studies have shown similar durations of viral shedding between symptomatic and asymptomatic cases [59], so we set $\rho _A^{-1}$ as four days as well. For the probability of developing symptoms, and the relative infectiousness of asymptomatic individuals, we use the CDC best estimate $q=0.6$ and $\delta =0.75$ [9]. The average stay in hospital is assumed to be 10 days, in accordance with the seven days median reported in [15]. The in-hospital death ratio ($\mu$) in the USA is 0.145 [10]. The best estimate for the infection fatality rate (IFR) is 0.0065 [9]; thus, the hospitalization probability $\eta$ of symptomatic cases can be inferred from the relation IFR $=q \eta \mu$ as $\eta \approx 0.076$.

**Table 1:** Parameters and values applied in the simulations

Parameter	Interpretation	Value	References
$R_0$	Basic reproduction number	2.2	[45]
$\alpha ^{-1}$	Latent period	2.5 (days)	[35]
$p^{-1}$	Pre-symptomatic infectious period	3 (days)	[2]
$\beta$	Transmission rate	1/3	Calculated
$\delta$	Relative transmissibility of asymptomatic	0.75	[9]
*q*	Prob. of developing symptoms	0.6	[9]
$\rho _I^{-1}, \rho _A^{-1}$	Infectious period	4 (days)	[2]
$\eta$	Hospitalization probability of symptomatic cases	0.076	[9]
$$h^{-1} $$	Average length of hospitalization	10 (days)	[15]
$\mu$	Probability of fatal outcome, given hospitalization	0.145	[10]
*N*	Population size (Hungary)	9,800,000	[34]

The basic reproduction number, expressing the average number of new infections generated by a single infected individual in a fully susceptible population, is given as

9 $$\begin{aligned} R_0=\beta \left( \frac{1}{p} +\frac{q}{\rho _I}+\frac{\delta (1-q)}{\rho _A}\right) . \end{aligned}$$

This formula can be derived as follows. Introducing a single infected individual into a susceptible population, then $S(t)/N \approx 1$. A newly infected individual, after passing through the latent phase, spends $p^{-1}$ time in the pre-symptomatic compartment, while infecting others with rate $\beta$. Then transits to the symptomatic infected compartment with probability *q*, where it spends $\rho _I^{-1}$ time infecting others again with rate $\beta$. Asymptomatic infection occurs with probability $1-q$, in which case the individual infects with reduced rate $\delta \beta$, for time $\rho _A^{-1}$ on average. Summing up these terms, we obtain (9). We assume that hospitalized individuals are properly isolated and do not cause significant numbers of infections.

Many studies have investigated $R_0$ for different countries; here, we use $R_0=2.2$ estimated from the Hungarian data [45]. From relation (9), given that all other parameters are determined, we can calculate $\beta =1/3$. We use Hungary’s population size for *N*. The parameter values are summarized in Table 1.

## The transmission dynamics model as a control system

To design a controller for the epidemic process, the first step is to define the manipulable parameters (control inputs) and identify the measured outputs. The latter comprises all relevant state-dependent variables that are available for measurement. In the absence of vaccination, one needs to rely on a variety of non-pharmaceutical measures, which are aiming to prevent the transmission of the virus. In our model, the control input, denoted by *u*, reflects the effect of the measures implemented to reduce the transmission rate. This variable is introduced in the model as a scaling factor of $\beta$, i.e., $\beta$ is replaced by $\beta (1-u)$ in Eqs. (1) and (2) which are therefore modified to

10 $$\begin{aligned} S'(t)= & {} - \beta (1-u(t))\left[ P(t) + I (t) + \delta A(t) \right] S(t)/N,\nonumber \\ \end{aligned}$$

11 $$\begin{aligned} L'(t)= & {} \beta (1-u(t)) [ P(t) + I (t) \nonumber \\&\,+\, \delta A(t) ] S(t)/N- \alpha L(t), \end{aligned}$$

where $0\le u(t) \le u_{\mathrm{max}} < 1,$ $\forall t\ge 0$. It is clear from the above equations that $u(t)=0$ corresponds to unmitigated disease spread without any restriction, and $u(t)=u_{\mathrm{max}}$ represents the strictest possible intervention level.

Analogously to $R_0$, the time-dependent effective control reproduction number, denoted by $R_c(t)$, can be given by

12 $$\begin{aligned} R_c(t)=\beta \left( 1-u(t)\right) \frac{S(t)}{N}\left( \frac{1}{p} +\frac{q}{\rho _I}+\frac{\delta (1-q)}{\rho _A}\right) .\nonumber \\ \end{aligned}$$

An analysis of eleven European countries [21] revealed that the reproduction number (3.6 on average) dropped to 0.66 after the strictest lockdowns; hence, we can assume $u_{\mathrm{max}}=0.82$.

**Table 2:** Typical measures applied in various countries

Banned visits to healthcare institutions and long-term care facilities
Suspension of flights, international travel restrictions
University and school closures
Shortened opening time of shops
Stay-at-home measures
Restriction of gatherings, cancel public events
Suspend public transportation
Test, trace, isolate
Closing non-essential businesses
Emergency notification
Public information and awareness campaign
Mask wearing requirements

**Fig. 2:**
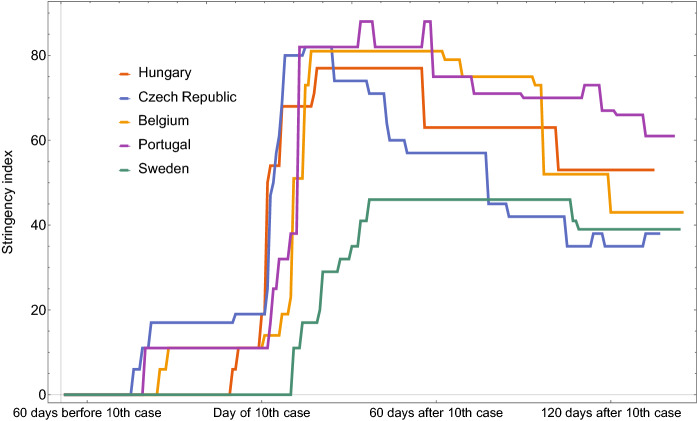
Stringency index of control measures in some countries of similar population sizes (Hungary, Czech Republic, Sweden, Belgium, Portugal). The data are taken from [24], and shifted in time to match the day of the 10th confirmed case in each country

### Realization of the control input by specific control measures

Public health authorities are implementing a wide range of measures in response to the COVID-19 outbreak; see Table 2. There exist recent works about the quantitative effect of different measures, usually in terms of the reduction of infection probabilities in different locations, e.g., in [51, 58]. These can be used to match input value ranges and various possible restrictions. The Oxford COVID-19 Government Response Tracker [24] is a tool that systematically collects information on several different common policy responses on 17 indicators such as school closures and travel restrictions. Such indicators can be composed into indices, such as the government response stringency index. Having data from more than 160 countries, one can rigorously track the evolving policy responses around the world and compare various countries. We have plotted the stringency index of selected European countries (that are similar to Hungary in population size) in Fig. 2. Later, we will see that the government responses of countries are very similar to constructed control inputs optimizing interventions with different cost functions and constraints.

Non-pharmaceutical measures aim to reduce the number of contacts between individuals or reduce the probability of transmission when contact is made. The transmission rate can be considered as

$$\begin{aligned} \beta= & {} \text {daily number of contacts}\\&\times \text {transmission probability}. \end{aligned}$$

Social distancing measures, such as school closures and banning of gatherings, reduce the average number of daily contacts made by an individual, while improved hygiene and mask wearing reduce the transmission probability. In our control system, we realize any combination of measures by changing $\beta$ to $\beta (1-u)$, where the control input *u* represents the overall effect of measures in reducing transmission. For example, if the number of contacts is reduced to half by social distancing measures, then $\beta (1-u)=0.5\beta$ thus $u=0.5$ If both the contact number and the transmission probability are reduced to half by a combination of measures, then the transmission rate is reduced to its quarter, corresponding to $\beta (1-u)=0.25\beta$, meaning that our control input is $u=0.75$.

### Discretization

The predictive control algorithm proposed in the next section requires a discrete-time dynamical model given in the general form $x_{k+1}=F(x_k,u_k)$. Therefore, the epidemic model (1) has to be discretized: function *F* has to be constructed s.t. $x_k\approx x(k\cdot T_s)$ for any piecewise constant input $u(t)=u_k$, $t\in [k\cdot T_s,(k+1)\cdot T_s)$, where $T_s$ is the sampling time and $x_k$ is a state vector. From the different possible discretization methods, we found that the simple forward Euler method is suitable for our purposes. It provides sufficient accuracy and preserves the structure of the continuous time model. We used a sampling time $T_s=0.5$ days to get the discrete time model for control synthesis. It is important to note that the discrete time model is used for control input design, but the actual trajectories of the system between the sampling instants are computed by an appropriate ODE solver using a standard explicit Runge–Kutta (4,5) method. In Sect. 5 a dynamic observer is designed for the epidemic model, which also requires a discrete time model. To increase the accuracy, that model is generated by a smaller ($T_s=0.1$ days) sampling time.

## Constrained state feedback control for mitigation

### Some relevant concepts from predictive control theory

In the first control scenarios, the entire state vector is assumed to be known. This assumption is not realistic, but the corresponding simulation results will show the physical limitations for controlling the epidemic process in the ideal situation when full information is available. In Scenario 6, this assumption will be relaxed and only the number of hospitalized COVID-19 patients (state *H* in the model) and the number of deceased (state *D* in the model) will be considered available.

In all scenarios, we design a feedback controller, i.e., the control input is periodically updated based on the actual measurements.

To formulate the control problem, the next step is to define the performance specifications that have to be satisfied by the controller and the controlled (closed-loop) system. The most criteria we expect from a conscious epidemic management can naturally be formulated by cost functions to be minimized (e.g., healthcare costs, or the harmful effects of restrictions on economy and society) and constraints to be satisfied (e.g., upper bounds for the number of hospitalized people and/or on the number of deaths). Model predictive control (MPC) methodology is therefore a promising approach for solving this problem. In the MPC framework, the control synthesis is transformed into a constrained optimization task solved in every discrete time step, when the control input has to be updated. Since the synthesis procedure boils down to a standard optimization problem, theoretically a wide set of possible cost functions and complicated constraints can be handled.

Formally, in case of discrete-time models and full state measurement, the main steps of the MPC algorithm can be summarized as follows:

1. A suitable control horizon $M\in {\mathbb {N}}_+$ is chosen; the time counter *k* is set to 0.
2. At time $k\cdot T_s$, state $x_k$ is measured. MPC is based on the prediction of the future states, therefore the following notation is introduced: the $(k+i)$th state predicted from the measurement made at time *k* will be denoted by $x_{k+i|k}$. By definition, $x_{k|k}=x_k$.
3. By applying the state update equation $x_{k+1}=F(x_k,u_k)$, the *M* predicted future states $\mathbf{x}_k=\{x_{k+1|k}$,$\ldots$,$x_{k+M|k}\}$ can be expressed as a function of the (yet unknown) future control actions $\mathbf{u}_k=\{u_{k|k},\ldots$, $u_{k+M-1|k}\}$. Using this formulation, an optimization problem can be defined:
  13a $$\begin{aligned}&~~~~~~~~~~\min _{\mathbf{u}_k} J(\mathbf{u}_k,\mathbf{x}_k) \end{aligned}$$
  13b $$\begin{aligned}&\text{ w.r.t. }~~ x_{k+i+1|k}=F(x_{k+i|k},u_{k+i|k}) \end{aligned}$$
  13c $$\begin{aligned}&~~~~~~~~~ G_x(\mathbf{x}_k)\le h_x,~G_u(\mathbf{u}_k)\le h_u \end{aligned}$$
  The objective function *J* and constraints (13c) are constructed to encode all design specifications to be satisfied by the controller and the closed-loop system. To solve (13), an appropriate numerical solver has to be used. The result is the optimal input sequence $\mathbf{u}_k^*=\{u_{k|k}^*,\ldots$, $u_{k+N-1|k}^*\}$.
4. The first element of $\mathbf{u}_k^*$ is applied to the process, i.e., $u_k:=u_{k|k}^*$. This control input is kept constant for $T_s$ time period. Then, *k* is incremented, i.e., $k:=k+1$, and the iteration continues at step 2.

We add the following important remarks to the MPC algorithm described above:

- In the description of the MPC above, we implicitly assumed that the system model is perfect: the model used for prediction is the same as that describes the true system behavior. In practical situations, this rarely holds: there are modeling uncertainties that may corrupt the prediction and thus the control input obtained. It is known that an appropriate feedback can significantly reduce the effect of uncertainties in itself [30, 48]. Moreover, there exist advanced methods for robust control synthesis and the robustness analysis of the closed loop. In the next section, no uncertainty is assumed for the model.
- The numerical complexity of the optimization problem depends on the structure of the cost functions and the constraints. Since the model is nonlinear, (13) becomes a nonlinear optimization problem. In the first control scenarios, we are going to investigate, quadratic cost function and linear constraints are used. Later, to formulate more complicated requirements, temporal logic constraints are also introduced, which turn the optimization task into a mixed integer nonlinear programming (MINLP) problem.
- The time horizon over which we intend to control the epidemic process is 180 days. We assume that the external conditions do not significantly change during this time period. Therefore, the behavior of the model beyond 180 days is not taken into consideration. (If further control is needed, new computations must be performed after 180 days.) Since the endpoint is fixed, the MPC is solved over shrinking horizon, i.e., *M* is time dependent and defined by $M_k=180-k$.
- If the entire state vector cannot be measured, the standard procedure is to augment the controller with a dynamical observer providing estimation for the true state. If the system is nonlinear, there is no general procedure for estimator design. This task can therefore be challenging: different existing methods have to be combined and adapted to the specific system model. In Sect. 5 we present a possible state estimator for the epidemic model above and show how it can be applied together with the MPC control.
- Although in the algorithm above the control input changes in every $T_s$ time period, this is not necessary: the frequency of control update can be easily decreased by simple constraints on *u*.

### Control scenarios

This section presents three control scenarios defined for the epidemic model. Each scenario addresses a different public health goal, and presents different control strategy. In all cases, full state measurement is assumed and all simulations start from the same initial condition: $S_0=N-L_0$, $L_0=40$, $P_0=I_0=A_0=H_0=D_0=R_0=0$, where *N* is the population of Hungary according to Table 1. We assume that the epidemic remains undetected until the number of hospitalized patients exceeds a small threshold $H_{thr}$. Technically, this means that the simulation runs open loop until this threshold is reached, the controller is switched on only thereafter. In the case studies we examined, $H_{thr}=10$ was used. As mentioned before, the sampling time is $T_s=0.5$ days, but in each scenario the control input is updated only weekly, i.e., in every 14th time instant. The simulations were run on a Dell Vostro 5471 computer with i7-8550U (4 cores, 1.8–4.0 GHz) processor and 8GB RAM under MATLAB R2019b using the BARON 19.3.24 solver [31] and YALMIP version R20200116 [36]. The code for the translation of specifications containing temporal logic expressions to optimization problems was based on the BluSTL toolbox [16].

#### Scenario 1: Mitigation and suppression with continuous control input

In this scenario, the control input is allowed to take arbitrary (continuous) values between 0 and an a priori defined $u_{\mathrm{max}}$. The cost function and constraints used in the MPC design are defined as follows:

14 $$\begin{aligned} \begin{aligned} J&=\sum _{i=0}^M u_{k+i|k}^2+w_HH_{M_k}+w_DD_{M_k}+w_\varepsilon \varepsilon , \\ H_{k+i+1|k}&\le {\overline{H}}+\varepsilon ,\quad 0\le \varepsilon , \quad 0\le u_{k+i|k}\\&\le u_{\mathrm{max}}, \quad \forall i=0\ldots M-1. \end{aligned} \end{aligned}$$

So, we would like to minimize the direct harmful effects of the restrictions (measured in a 2-norm), and keep the number of hospitalized patients under a predefined upper bound not to overload the healthcare system. The weighting factor $w_D$ penalizing the number of deceased at the end of the horizon can be used to balance between *mitigation* and *suppression*, the two typical goals of COVID-19 management [20]. In the first case, $w_D=w_H=0$, so the focus is only on the direct cost of the control measures. The controller is expected to avoid strict measures and thus only mitigates the effects of the epidemic to the extent that the hospitalization remains below the given bound. In the second case, $w_D\gg 0$, $w_H\gg 0$ are set such that the corresponding terms in the cost function are comparable with $\sum _{i=0}^M u_{k+i|k}^2$, so the controller tries to suppress the epidemic even if the control actions are expensive (i.e., they have harmful effects). The upper bound ${\overline{H}}$ represents the limit of the healthcare capacity. Parameters $w_\varepsilon$ and $\varepsilon$ are the ingredients of the soft constraint formulation. Soft constraint is applied to avoid the possible numerical infeasibility that can occur in the vicinity of ${\overline{H}}$ by the slight difference between the simulated continuous and the predicted discrete trajectories.

**Fig. 3:**
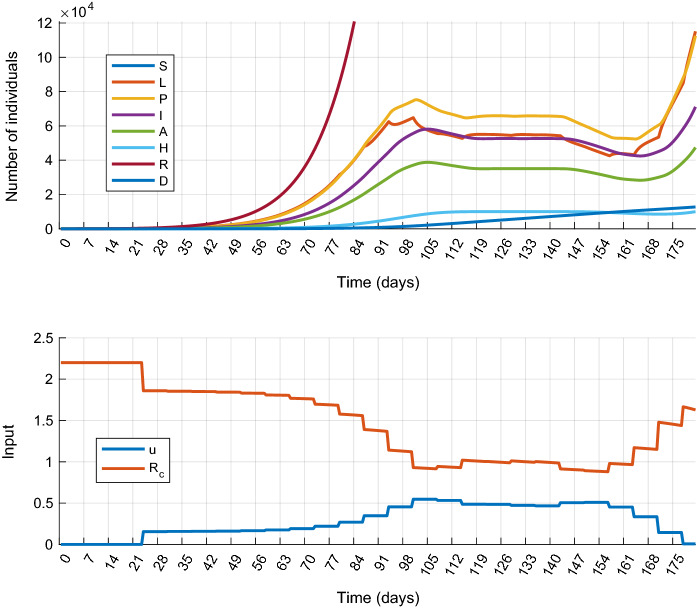
Simulation results of Scenario 1.a (Mitigation): state trajectories (top) and control inputs with the corresponding effective reproduction number $R_c$ (bottom)

First the *mitigation* scenario is investigated. For this, simulations have been performed with the following parameter values: $\overline{H}=10{,}000$, $u_{\mathrm{max}}=0.82$. The results obtained are shown in Fig. 3. At the beginning of the control period the control input is small. This shows that less strict measures are sufficient during this time. As the epidemic progresses the control input slowly increases, but only until the 98th day, when it reaches a higher but still moderate value that is significantly smaller than the allowed maximum $u_{\mathrm{max}}$. After the 98th day, the epidemic can be successfully mitigated. At the end of the control period (from day 154) the controller eases the restrictions (the control input decreases) since the control specifications have to be fulfilled only up to the 180th day, and this can be achieved even if the measures are relaxed (the control cost is decreased) in the last few weeks. If the constraints have to be satisfied on a longer time period, the control horizon has to be increased. From this result, the following conclusion can be drawn: first, if we can intervene in time, there is no need to immediately implement strict measures, and second, the epidemic can be mitigated by applying only moderate restrictions. The total cost of the control strategy is $J_{1m}^*=42.86$.

**Fig. 4:**
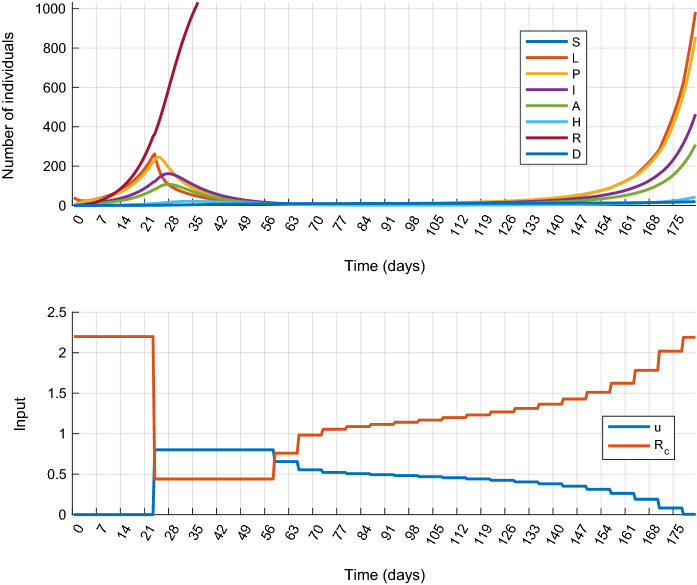
Simulation results of Scenario 1.b (Suppression): state trajectories (top) and control inputs with the corresponding effective reproduction number $R_c$ (bottom)

It is important to notice the increase of the state variables at the end of the horizon. Since finite time control policy is computed, it is not surprising that close to the end of the control period, the controller decreases the control input to minimize the cost. As a response, the state variables start to increase, but this does not cause feasibility problem as long as the constraints are not violated till the end of the horizon. This so-called turnpike behavior shows that easing the measures would result in an epidemic peak. With strict constraint on the healthcare capacity, this could be satisfactorily avoided only if a suitable herd immunity is reached by the end of the control horizon. It has been documented in several papers, e.g., [26, 32] that in case of COVID-19 pandemic, to reach herd immunity without overwhelming the healthcare system would take years. Consequently, defining a good terminal constraint for this relatively small time period is not possible. What can be done is to directly constrain the increase of the states at the final (*M* and $M-1$-th) time instants [32]. We are going to show an example for this in Scenario 3.

**Fig. 5:**
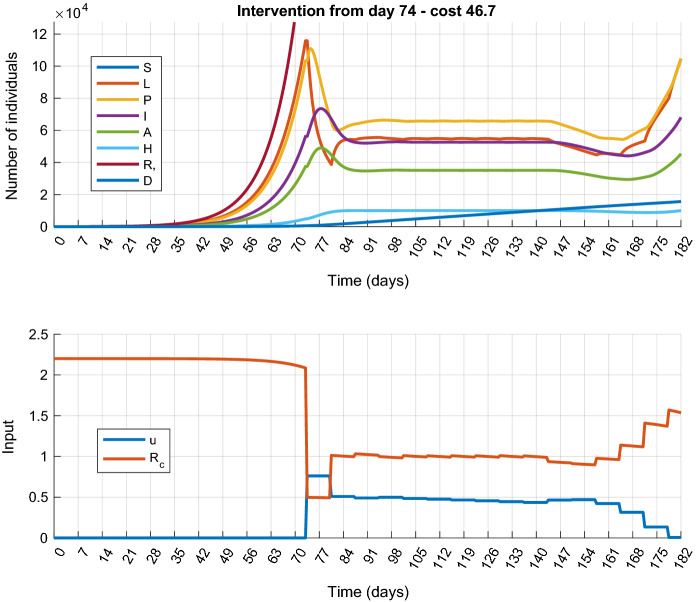
Simulation results of Scenario 1.a with delayed intervention. Simulation results obtained at the maximal tolerable input delay ($d=74$ days)

Using the mitigation setup we have analyzed the maximal delay that the system can tolerate before implementing any measure. From a control perspective, this means that the system runs open loop (i.e., uncontrolled) in the time interval $[0,d\cdot T_s]$, where $d\in {\mathbb {N}}_+$ and then the controller is turned on. We seek the maximal *d*, for which the MPC optimization problem has a feasible solution. For the maximal tolerable delay, we have obtained 74 days (i.e., $d=144$). For larger values of *d*, the MPC optimization has no feasible solution. (To detect infeasibility, a hard upper bound has been introduced for the soft constraint violation. Specifically, in this scenario, $\varepsilon \le 0.01$ has been used.) The simulation results are plotted in Fig. 5. Considering the control input, it can be seen that as expected, the larger the delay the stricter the measures that have to be applied. The maximal control input is 0.82, which corresponds to total lockdown.

The controller for epidemic *suppression* has been designed by the following weights in the cost function: $w_D = 0.0267$ and $w_H = 0.0033$. The simulation results are plotted in Fig. 4. It is visible that the outbreak can be successfully suppressed for the price of a strict and early lockdown, followed by a slow gradual easing of the measures. However, a second wave of the epidemic appears at the end of the horizon as it has been observed in several countries, for example, the curves in Fig. 4 show a striking resemblance to the true epidemic curve of Hungary . The total cost of the control strategy is $J_{1s}^*=101.8$ from which the cost of the control input is $\sum _{k}u_k^2=89.27$.

#### Scenario 2: The effect of control input quantization

By definition, the control input *u* reflects the effect of different measures implemented by the government in the society. Since there is a finite number of measures that can be applied (Table 1), a control input with truly continuous range cannot be realized in practice. Motivated by this, we assume now that the control input is quantized and can take only 4 different values. Each value corresponds to a specific measure as follows: $u^{(1)}=0,u^{(2)}=0.19,u^{(3)}=0.41,u^{(4)}=0.6$. Here, as an example, $u^{(2)}$ may correspond to school closures, $u^{(3)}$ to stay-at-home orders, and $u^{(4)}$ can be interpreted as a combination of the two. To force $u_k\in \{u^{(1)},u^{(2)},u^{(3)},u^{(4)}\}$ for all *k*, an additional constraint is added to the MPC synthesis:

15 $$\begin{aligned} \square (u=u^{(1)} \vee u=u^{(2)} \vee u=u^{(1)} \vee u=u^{(4)}), \end{aligned}$$

where $\square$ is a temporal logic operator called “always” and is defined as follows: if $\phi$ is an arbitrary logical expression, then

16 $$\begin{aligned}&\square _{[a, b]} \phi \text { is true at time } t \nonumber \\&\qquad \Leftrightarrow ~~\forall t' \in [t+a, t+b] \text { the formula } \phi \text { is true.} \end{aligned}$$

Using this definition, constraint (15) prescribes that one of the four equations $u=u^{(i)}$, $i\in \{1,2,3,4\}$ has always to be true. (More details on temporal logic operators can be found, e.g., in [18]). We remark that the discrete inputs alone do not necessitate the application of temporal logic (see, e.g., [37]). However, this notation is intuitive, and using the temporal logic framework it is straightforward to add more complex (possibly time-varying) constraints as it will be shown by the next scenario.

**Fig. 6:**
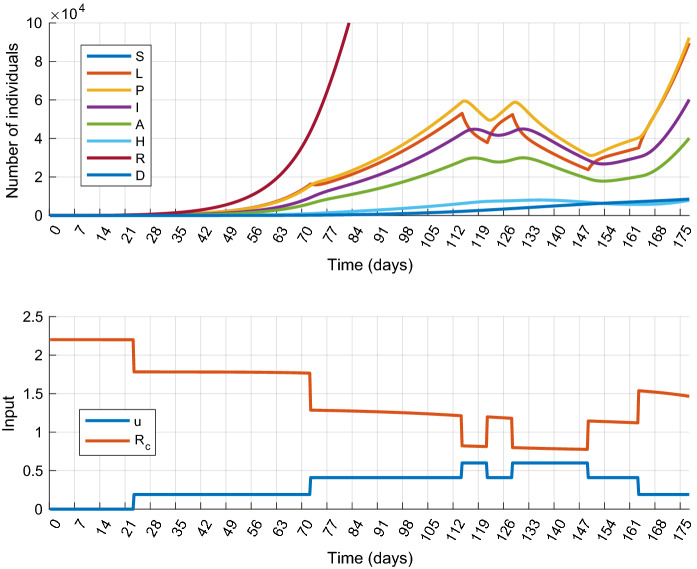
Simulation results of Scenario 2 (Control input quantization): state trajectories (top) and control inputs with the corresponding effective reproduction number $R_c$ (bottom)

To analyze the effect of input quantization, we have performed the mitigation scenario defined in the previous section with the additional constraint (15). The results are plotted in Fig. 6. It can be seen that the primary control goal, i.e., the mitigation of the epidemic is achieved and the input and state constraints are satisfied. It is also important to mention that the quantized control input is similar to the continuous one obtained in Scenario 1, which means that the optimal control strategy is very similar in the two cases. On the other hand, the quantization allows less freedom to the controller, so the total cost is now higher: $J_2^*=45.88$.

#### Scenario 3: Refined constraint for healthcare capacity

In this scenario we allow, but only once and only for a limited time period, that the number of hospitalized patients (*H*) exceeds the limit $\overline{H}$. This scenario represents the case when there is an extra, but possibly costly reserve in the healthcare system that can be activated if necessary, or resources are reallocated to COVID-19 from other areas of healthcare. Formally, we introduce two new parameters: $T_{r}$ and $\overline{\overline{H}}$, such that $\overline{H}<\overline{\overline{H}}$ and the MPC design is completed with the following constraint:

17 $$\begin{aligned} \square (H \le \overline{H}) ~ \mathbf{U }\left( \square _{[0,~T_r]} (H \le \overline{\overline{H}}) \wedge \square _{[T_r,~N]} (H\le \overline{H})\right) \end{aligned}$$

where the temporal logic operator $\mathbf{U }$ (called “until”) is defined as follows:

18 $$\begin{aligned}&\varphi \mathbf{U }_{[a, b]}\psi \text { is true at time }t \nonumber \\&\qquad \Leftrightarrow ~~ \exists t' \in [t+a, t+b] \text { st. } \psi \text { is true} ~~ \wedge ~~ \end{aligned}$$

19 $$\begin{aligned}&~~ \forall t'' \in [t, t'] \,\varphi \text { is true} \end{aligned}$$

**Fig. 7:**
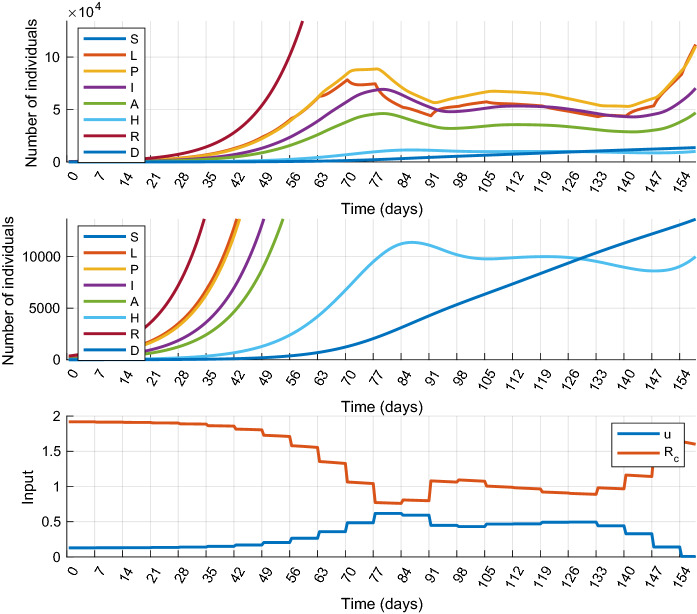
Simulation results of Scenario 3 (Temporal increase of healthcare capacity): state trajectories (top) and control inputs (bottom) obtained with $T_r=21$ and $\overline{\overline{H}}=15{,}000$. *H* is above 10,000 between days 79 and 100

**Fig. 8:**
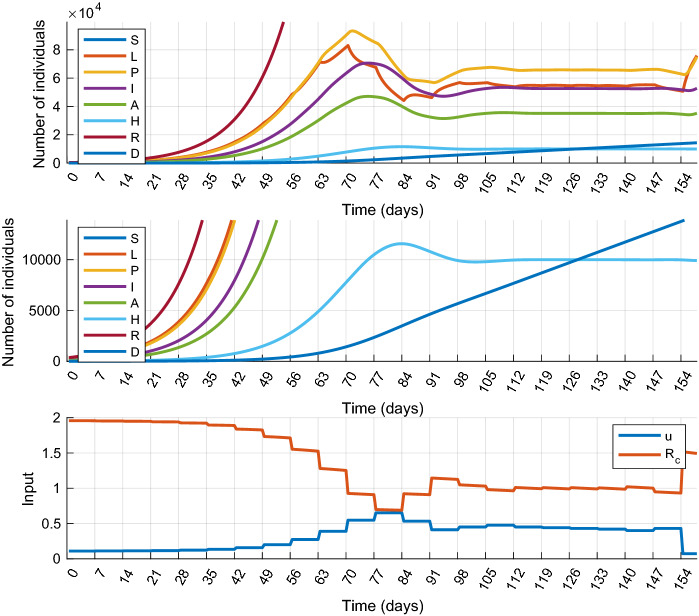
Simulation results of Scenario 3 (Temporal increase of healthcare capacity): state trajectories (top) and control inputs (bottom) obtained with $T_r=21$ and $\overline{\overline{H}}=15{,}000$. In this simulation, a terminal constraint for the number of hospitalized individuals has also been introduced. *H* is above 10,000 between days 76 and 97

In expression (17), $\overline{\overline{H}}$ denotes a new upper bound that is never to be violated and $T_r$ is the maximal time period for which $H>\overline{H}$ is allowed. The numerical simulation for this scenario was performed with the following parameter values: $\overline{\overline{H}}=15{,}000$ and $T_r=21$ days. The results obtained by performing a mitigation scenario are depicted in Figs. 7 and 8, respectively. Compared to the results of Scenario 1, it can be seen that the shapes of the control inputs are similar. The main difference is that the controller in Scenario 3 applies smaller control actions over almost the entire horizon. The control input is larger only for a short period after $T_r$ is elapsed. This is necessary to stop the increase of the constrained state variables, which would result in the violation of the constraints and the loss of feasibility. Since the control input is smaller at most times than in Scenario 1, the total cost of the control is smaller: $J_{1m}^*=42.86$ in Scenario 1 and $J_3^*=41.43$ in Scenario 3.

Similar to the other scenarios investigated so far, the state variables start to increase at the end of the control horizon. To avoid this behavior, we introduce the following simple terminal constraint:

20 $$\begin{aligned} H_{k+M|k}+1\le H_{k+M-1|k} \end{aligned}$$

i.e., the number of hospitalized individuals must decrease in the last step. This constraint prevents *H* and the other states from increasing: strict control measures are applied till the very end of the horizon. Though the characteristic of the state variation has been significantly improved, nothing can be guaranteed for the process behavior beyond the control horizon. A later outbreak can be avoided only if the implementation of the carefully planned, strict control policy is continued.

## State estimator design and output feedback control

In this section, the assumption of full state measurement is dropped, and aligned with the common practice, only the number of the deceased (*D*) and the number of the hospitalized individuals (*H*) are monitored. There are examples in the COVID-19 literature, where the global dynamics and the epidemic curve was reconstructed from the data of hospitalized or deceased individuals [23, 43]. In order to use the state feedback MPC controller, a dynamical observer is designed to estimate the remaining non-measured states.

### LPV observer design for the epidemic model

To design the estimator, the states are normalized first and the dynamical model is divided into three parts. According to the three subsystems, the state vector is partitioned as follows: $s:=S/N$, ${\bar{x}}=[L,P,I,A,H]/N$ and $r=R/N$. Focusing on *x*, we notice that the corresponding dynamical equations can be rewritten in linear parameter-varying (LPV) form:

$$\begin{aligned} {\bar{x}}_{k+1} = (I+T_sA_0+\rho _k T_sA_1){\bar{x}}_k\doteq A(\rho _k){\bar{x}}_k \end{aligned}$$

where $\rho _k=s_kv_k$ with $v_k=1-u_k$ is the scheduling variable and

21 $$\begin{aligned} A_0&=\left[ \begin{matrix} -\alpha &{}\quad 0 &{}\quad 0 &{}\quad 0 &{}\quad 0\\ \alpha &{}\quad -p &{}\quad 0 &{}\quad 0 &{}\quad 0 \\ 0 &{}\quad qp &{}\quad -\rho _I &{}\quad 0 &{}\quad 0 \\ 0 &{}\quad (1-q)p &{}\quad 0 &{}\quad -\rho _A &{}\quad 0 \\ 0 &{}\quad 0 &{}\quad \rho _I\eta &{}\quad 0 &{}\quad -h \end{matrix}\right] ,\nonumber \\ A_1&=\left[ \begin{matrix} 0 &{}\quad \beta &{}\quad \beta &{}\quad \delta \beta &{}\quad 0 \\ 0 &{}\quad 0 &{}\quad 0 &{}\quad 0 &{}\quad 0 \\ 0 &{}\quad 0 &{}\quad 0 &{}\quad 0 &{}\quad 0 \\ 0 &{}\quad 0 &{}\quad 0 &{}\quad 0 &{}\quad 0 \end{matrix}\right] \end{aligned}$$

follow from (1). By introducing $C=[0~0~0~0~1]$, a measurement equation is added to the model: $y_H=C{\bar{x}}$, where $y_H={\bar{x}}_5=H/N$. Assume $\rho$ is bounded, i.e., $\rho \in [{\underline{\rho }},~{\overline{\rho }}]$ and ${{\underline{\rho }}}$, ${{\overline{\rho }}}$ are a priori known. If we assume that up to half of the population gets infected, then $s\in [0.5,1]$ holds. This together with the input constraint $u\in [0,~0.7]$ gives the bound for $\rho$: $\rho \in [0.15,1]$. Using these bounds, a parameter-varying observer can be designed, but in order to use it, the scheduling variable ($\rho$) has to be known at each time instant. Since in our case $s_k$ is not available for measurement, we can only approximate it by using its difference equation, as follows:

22 $$\begin{aligned} \hat{s}_{k+1}={\hat{s}}_k-T_s{\hat{s}}_k v_k \left[ 0~-\beta ~-\beta ~-\beta \delta ~0\right] \hat{ {\bar{x}}} \end{aligned}$$

By scheduling the model with ${\hat{\rho }}={\hat{s}}v$, we face the problem of *observer design for LPV systems with inaccurately measured scheduling variables*. This problem is well identified in control literature and one possible solution is proposed in [11, 25, 38]. The papers discuss different variants, namely differently improved versions of the same approach introduced first in [38]. The method constructs a parameter-varying observer, scheduled by ${\hat{\rho }}$ such that the boundedness of the estimation error is guaranteed as long as $\rho -{\hat{\rho }}$ is bounded.

Before applying this method, it is important to check the observability properties of the LPV model. The quickest analysis is to compute the observability matrix at different frozen (fixed) parameter values. This is a necessary condition for the parameter-dependent observability. Taking 10 equidistant points $\rho _1\ldots \rho _{10}$ on the interval [0.15, 1], we have found that the linear time-invariant (LTI) models $(A(\rho _i),C)$ are all observable: the corresponding observability matrices have full rank. However, it is important to note that these matrices are badly conditioned, they are close to singular, so the model is only weakly observable. This may challenge the observer design process and has effect on the achievable performance of the state estimation. It is also important to keep in mind that while the properties of the LPV model can give information on the properties of the nonlinear system, the two systems are not the same: the epidemic model is embedded in the LPV structure, so the latter describes a much broader dynamical behavior.

Starting from the LPV model, the state estimator is defined in the following form:

23 $$\begin{aligned} \hat{{\bar{x}}}_{k+1}=A({\hat{\rho }}){\hat{\bar{x}}}_k+L({\hat{\rho }})(y_H-{\hat{y}}_H) \end{aligned}$$

where ${\hat{y}}_H=C{\hat{x}}$. This results in the following error dynamics:

24 $$\begin{aligned} e_{k+1}={\bar{x}}_{k+1}-{\hat{\bar{x}}}_{k+1}=(A({\hat{\rho }})-L({\hat{\rho }})C)e_k+\gamma _k \end{aligned}$$

where $\gamma _k=(A(\rho _k)-A({\hat{\rho }}_k)){\bar{x}}_k$. By fixing the feedback gain $L({\hat{\rho }})$ in parameter affine form $L_0+{\hat{\rho }} L_1$, the coefficient matrices $L_0$ and $L_1$ can be determined by finding positive definite $P_i$ and general $G_i$, $F_i$ matrices for $i\in \{1,2\}$ that satisfy the following linear matrix inequalities (LMI):

25 $$\begin{aligned} \begin{aligned} \left[ \begin{matrix} P_i &{} \quad A_i^{{\mathrm{T}}}G_i-C^{{\mathrm{T}}}F_i^{{\mathrm{T}}} \\ G_iA_i-F_iC &{} \quad G_i^{{\mathrm{T}}}+G_i-P_j \end{matrix}\right]&\succ \quad 0, ~ i,j\in \{1,2\},\\ A_1&=A({\underline{\rho }}), ~A_2=A({\overline{\rho }}). \end{aligned} \end{aligned}$$

Then with ${\bar{L}}_i=G_i^{-1}F_i$, the observer gains are computed as follows: $L_1=1/({{\overline{\rho }}}-{{\underline{\rho }}})({\bar{L}}_2-\bar{L}_1)$, $L_0={\bar{L}}_1-{{\underline{\rho }}} L_1$. It is shown in [38], that the dynamics of the estimation error (24) is input-to-state stable (ISS) with respect to input $\gamma _k$. This implies that $e_k\rightarrow 0$ as $k\rightarrow \infty$ if $\gamma _k=0$ (i.e., $\rho _k={\hat{\rho _k}}$) and also that $e_k$ is bounded for all *k* if $\rho _k-{\hat{\rho _k}}$ is bounded. Note that the observer design procedure considers the scheduling parameter independent of the state prediction. Formally this is true, as $\rho _k$ depends on $s_k$ which is not element of ${\bar{x}}_k$. Thinking in this way, the design is correct and the properties of the LPV observer can be independently analyzed: for example, a bound for the ISS gain can be computed for (24) by using [38]. On the other hand, in our specific setup the dynamic equation (22) couples ${\hat{\rho }}$ and ${\bar{x}}$. This makes the analysis of the observer more challenging. We therefore make the further analysis via simulations by interconnecting the observer, the dynamics of ${\hat{s}}$ and the nonlinear system model.

In the possession of ${\hat{s}}$ and ${\hat{x}}$, the remaining state variable *r* can be obtained by iterating its state update equation:

$$\begin{aligned} {\hat{z}}_{k+1}=z_{k}+T_s[0~ 0~ \rho _I(1-\eta )~ \rho _A~ (1-\mu )h]\hat{{\bar{x}}}_k \end{aligned}$$

Note, ${\hat{r}}$ is thus constructed by integrating the linear combination of the other states. We cannot prove anything for the boundedness of $z-{\hat{z}}$, but this is not a serious issue as *r* does not influence the behavior of the other states and it is used only in a control objective of Scenario 4. Since a lower limit for the number of infected patients is not a strict value, some deviation from the prescribed limit is not critical. Simulations will, however, reveal that $z-{\hat{z}}$ is actually small over the control horizon, so ${\hat{z}}_k$ is a suitably precise estimate for $z_k$. It is also important to mention that measurement *D* is not used in the observer. Since *D* does not influence the other state variables, measuring it is not relevant to the observer design (but it is very useful to precisely evaluate the cost function). It has to be admitted that the assumption of precisely knowing the model parameters is not completely realistic. Therefore, tracking the number of hospitalized people only may not be enough in practice to compute the population in other compartments with the required precision. To address this problem, the effect of parameter uncertainty for a controller–observer configuration is examined later in Sect. 5.4.

### Numerical results obtained by the LPV observer

By solving (25), the following observer gains have been obtained:

26 $$\begin{aligned} L_0=\left[ \begin{matrix} 13.4913 \\ 14.1086.\\ 8.3603 \\ 5.5759 \\ 1.0058 \end{matrix}\right] ,\quad L_1=\left[ \begin{matrix} 1.3190 \\ 0.0767 \\ -\,0.0009 \\ -\,0.0019 \\ 0.0001 \end{matrix}\right] . \end{aligned}$$

However, due to the weak observability, the error dynamics is close to the boundary of stability, the matrices $P_1$, $P_2$ characterizing the Lyapunov function are numerically ill-conditioned: there are several orders of magnitude difference between their eigenvalues. Further analysis is thus necessary to reveal the performance properties of the observer, e.g., to compute an upper bound for the magnitude of the estimation error. Papers [11, 25] refine the algorithm above and derive such a performance metric. In this paper, we cannot go into the details of this analysis procedure, we examine the observer only in numerical simulations and place the focus on its application in closed-loop control.

**Fig. 9:**
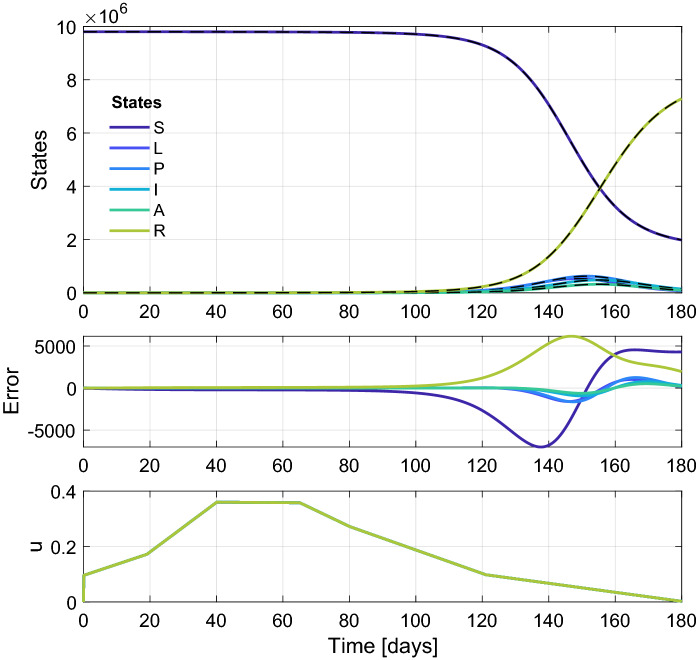
True (colored solid line) and estimated states (dashed black line) (top figure) and estimation error (middle figure) obtained by the state observer. The control input applied during the simulation is plotted in the bottom figure. (Color figure online)

Figure 9 presents the simulation results obtained by running the system open loop with the control input depicted in the same figure. The initial state was the same as we chosen above, i.e., $L_0=40$, $S=N-L$, and the other states are 0. In the simulation, the normalized states were estimated, but they were rescaled to plot the results. It can be seen that noticeable, but still not significant estimation error can be detected only in variables *S* and *R* and only in the neighborhood of the peak of the epidemic. This is not relevant, however, since the estimator is intended to be used together with a controller, which mitigates or suppresses the epidemic peak.

### Scenario 4: Output feedback control

**Fig. 10:**
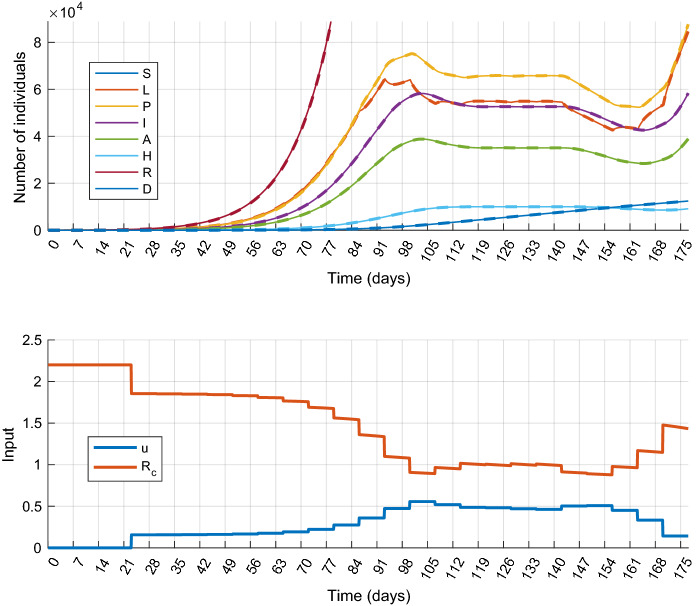
Simulation results of Scenario 4 (Output feedback control): state trajectories (top) and control inputs with the corresponding effective reproduction number $R_c$ (bottom). The true and estimated state trajectories are plotted by colored solid and black dashed lines, respectively. (Color figure online)

In this section, we examine how the state observer works together with the MPC controller. For this, we repeat the simulation of Scenario 1 (Sect. 4.2.1) with the following modification: the precise state measurement $x_k$ is replaced by the estimated value ${\hat{x}}_k$. The simulation results are plotted in Fig. 10. The control input and state trajectories obtained in the two scenarios can hardly be distinguished. Since the epidemic peak, where the estimation error would be noticeable, is mitigated, the state estimation is almost perfect over the entire horizon. Consequently, using ${\hat{x}}_k$ in the control input computation has only negligible effect on the closed-loop behavior. Compared to Scenario 1, the control costs are almost equal in the two scenarios: $J_1^*=42.86$, $J_4^*=42.98$. We can conclude that the lack of direct measurement of *S* is not crucial from the point of view of state measurement if the observer is used in closed-loop control.

### Scenario 5: Effect of parameter uncertainty

We have assumed so far that the dynamical model of the epidemic is precisely known, that is the model (1)–(8) with parameters in Table 1 accurately describes the dynamical behavior of the epidemic process. This is hardly the case in a real situation. Therefore, the possible parameter uncertainties have to be taken into account during the control design process. This leads to a robust synthesis, which is beyond the scope of this paper. On the other hand, to study the applicability of the proposed control method, it is important to examine how it works in the presence of model mismatch. In this subsection, we show several simulations with the output feedback scenario presented above with the following settings: the model structure used for prediction and state observation is the same, but certain parameters of the controlled system are different in each experiment. We assume that four parameters, namely $\alpha ,q,\delta ,\eta$ are uncertain, they take values from the following intervals:

27 $$\begin{aligned} \alpha&\in [1/3,~1/2],\quad \delta \in [0.7,~0.8],\quad q\in [0.5,~0.7],\nonumber \\&\eta \in [0.069,~0.083], \end{aligned}$$

**Fig. 11:**
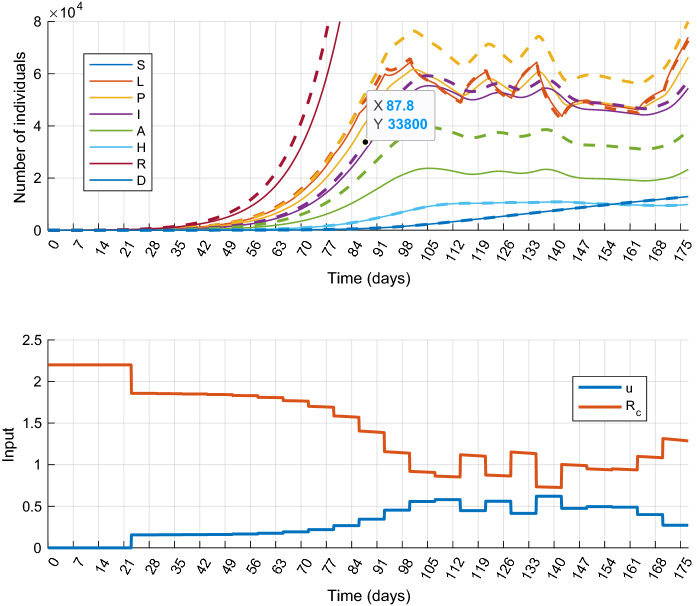
Simulation results of Scenario 5 (Output feedback control) with model uncertainties. State trajectories (top) and control inputs with the corresponding effective reproduction number $R_c$ (bottom) in case 7 of Table 3 The true and estimated state trajectories are plotted by colored solid and black dashed lines, respectively. (Color figure online)

**Fig. 12:**
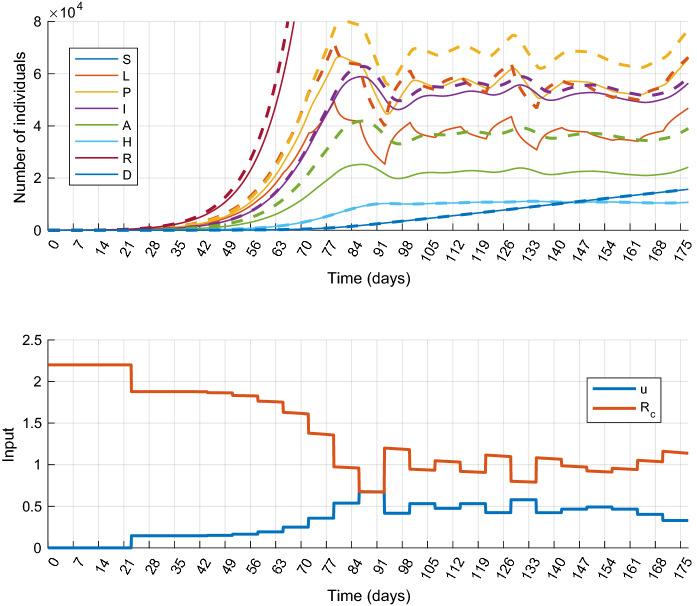
Simulation results of Scenario 5 (Output feedback control) with model uncertainties. State trajectories (top) and control inputs with the corresponding effective reproduction number $R_c$ (bottom) in case 15 of Table 3. The true and estimated state trajectories are plotted by colored solid and black dashed lines, respectively. (Color figure online)

The upper and lower bounds of the parameter domains have been determined using the references in Table 1. Further, we assume that the other model parameters are more precisely known, and therefore their nominal values were used in the simulations. We remark that possible uncertainty in $\beta$ can be handled, since due to the model structure, designing for larger $\beta$ gives a feasible controller for smaller values as well. To analyze the robustness, 16 simulations defined by the possible combinations of the min–max values of the uncertain parameters have been performed. Table 3 collects the parameters of the experiments with the results obtained. The detailed simulation results obtained for cases 7 and 15 are plotted in Figs. 11 and 12, respectively.

It can be seen that the controller worked acceptably well with uncertain models, although the cost varied visibly for the different cases. Regarding the constraint on the healthcare capacity, it is only violated in half of the simulations and the transgression of the limit is not critical. On the other hand, there is a room for performance improvement, and thus improving the robustness of the controller is an important task in the future.

**Table 3:** Model parameters of the experiments performed for uncertainty analysis

Case number	${\alpha ^{-1}}$	${\delta }$	*q*	${\eta }$	Cost	maxH
0	3.00	0.70	0.50	0.69	33.54	9305
1	3.00	0.70	0.50	0.83	35.33	9608
2	3.00	0.70	0.70	0.69	41.22	10,290
3	3.00	0.70	0.70	0.83	43.79	10,504
4	3.00	0.80	0.50	0.69	36.86	9738
5	3.00	0.80	0.50	0.83	38.96	9939
6	3.00	0.80	0.70	0.69	43.38	10,781
7	3.00	0.80	0.70	0.83	46.30	10,893
8	2.00	0.70	0.50	0.69	37.37	9336
9	2.00	0.70	0.50	0.83	39.65	9545
10	2.00	0.70	0.70	0.69	46.03	10,242
11	2.00	0.70	0.70	0.83	49.20	10,765
12	2.00	0.80	0.50	0.69	40.24	9691
13	2.00	0.80	0.50	0.83	44.64	9834
14	2.00	0.80	0.70	0.69	48.28	10,651
15	2.00	0.80	0.70	0.83	51.70	11,105

## Discussion

The model-based control of the spread of the COVID-19 epidemic was proposed in this paper. The dynamical model is given in the form of a set of nonlinear ODEs containing eight compartments. The model parameters were determined from the literature and the epidemic data recorded in Hungary between March and May 2020. The assumed manipulable control input with strict upper and lower bounds is the time-varying transmission rate affected by different restrictive measures planned and implemented by the authorities to slow down disease spread.

A model predictive control approach was proposed which uses the discrete-time version of the dynamical model and is able to take into consideration complex specifications and constraints containing even integer variables and logical relations in the form of temporal logic expressions. The control goals are then automatically translated to a MINLP problem, capable of handling the nonlinear system dynamics. To address the realistic situation when not all state variables are observed continuously, a state observer is proposed using the theory of LPV systems, assuming that only the number of hospitalized and deceased patients are known on a daily basis. In the numerical simulations, we found that the number of people in the other 6 compartments can be computed with sufficient precision using the observer if the model parameters are known, although the model itself is numerically only weakly observable due to the possible different orders of magnitudes of the susceptible and the infected population. This underlines the importance of regularly tracking the susceptible population (which is a scheduling parameter in the state observer), since the online estimation of the other states could be significantly improved by that. In practice, this can be achieved by large scale serological surveys.

Five control scenarios were shown and analyzed with different goals and assumptions. The scenarios cover the well-known cases of mitigation, when the direct cost of the intervention (control) is minimized with a constraint on healthcare capacity, and also suppression, where the cost is assigned to infection, hospitalization and fatalities. It is worthwhile to note that there is a striking resemblance between the constructed control inputs and real-life government responses, measured by a stringency index, both for mitigation and suppression strategies. We have also monitored the time-varying effective reproduction number $R_t$, which became a very popular measure of the current epidemic situation during the COVID-19 pandemic. For suppression, we see that very strict measures (lockdown) are necessary initially, and they can be slowly relaxed later. This corresponds to a sharp drop in $R_t$ to levels way below one. On the other hand, for mitigation, the stringency of the control is increased much more slowly, and maximized at a moderate level, while $R_t$ is being kept around the critical value 1 for a long time period.

We emphasize that the proposed flexible approach is able to directly handle predefined discrete levels of restrictions. The output feedback design case (i.e., the combination of the controller and observer) was also examined through several simulations assuming uncertainties in selected parameters. It is justified by the computational results that an early intervention is of key importance in satisfying the control goals and constraints. The feasibility analysis corresponding to the model predictive control problem is also useful to assess the practical (physical, biological) limits of the planned interventions and identify late actions. Future work will be focused on the sensitivity and further robustness analysis of the approach and on the specification of even more realistic goals and constraints. Among the latter, assigning individual costs to different types of restrictions (Table 2) and putting the optimal selection between them into the framework of optimal control may add further value to the research.

## References

[1] Ames, A.D., Molnar, T.G., Singletary, A.W., Orosz, G.: Safety-critical control of active interventions for COVID-19 mitigation. medRxiv (2020). 10.1101/2020.06.17.2013326 10.1109/ACCESS.2020.3029558 PMC8545284 34812361

[2] Ashcroft, P., Huisman, J.S., Lehtinen, S., Bouman, J.A., Althaus, C.L., Regoes, R.R., Bonhoeffer, S.: COVID-19 infectivity profile correction. Preprint (2020). arXiv:2007.06602 10.4414/smw.2020.20336 32757177

[3] Barbarossa, M.V. et al.: A first study on the impact of current and future control measures on the spread of COVID-19 in Germany. medR$$\chi $$iv 2020.04.11.10.1101/2020.04.08.20056630

[4] Becker NG (2015). Modeling to Inform Infectious Disease Control.

[5] Belta C, Yordanov B, Gol EA (2017). Formal Methods for Discrete-Time Dynamical Systems.

[6] Boldog P, Tekeli T, Vizi Zs, Dénes A, Bartha FA, Röst G (2020). Risk assessment of novel Coronavirus COVID-19 outbreaks outside China. J. Clin. Med..

[7] Böhmer MM (2020). Investigation of a COVID-19 outbreak in Germany resulting from a single travel-associated primary case: a case series. Lancet Infect. Dis..

[8] Bussell EH, Dangerfield CE, Gilligan CA, Cunniffe NJ (2018). Applying optimal control theory to complex epidemiological models to inform real-world disease management. Philos. Trans. R. Soc. B.

[9] CDC COVID-19 Pandemic Planning Scenarios: US CDC. https://www.cdc.gov/coronavirus/2019-ncov/hcp/planning-scenarios.html

[10] COVID-NET: A weekly summary of US COVID-19 Hospitalization Data. https://gis.cdc.gov/grasp/COVIDNet/COVID19_5.html

[11] Daafouz, J., Millerioux, G., Rosier, L.: Observer design with guaranteed bound for LPV systems. In: IFAC World Congress, pp. 107–112 (2005)

[12] de la Sen M, Alonso-Quesada S (2011). Vaccination strategies based on feedback control techniques for a general SEIR-epidemic model. Appl. Math. Comput..

[13] Deslandes A (2019). SARS-CoV-2 was already spreading in France in late. Int. J. Antimicrob. Agents.

[14] Di Domenico, L., et al.: Expected impact of lockdown in Île-de-France and possible exit strategies. medR$$\chi $$iv (2020). 10.1101/2020.04.13.20063933

[15] Docherty, A.B., et al.: Features of 16,749 hospitalised UK patients with COVID-19 using the ISARIC WHO clinical characterisation protocol. medR$$\chi $$iv (2020). 10.1101/2020.04.23.20076042

[16] Donzé, A., Raman, V.: BluSTL: controller synthesis from signal temporal logic specifications. In: 1st and 2nd International Workshop on Applied Verification for Continuous and Hybrid Systems. EPiC Series in Computer Science, vol. 34, pp. 160–168 (2015)

[17] ECDC: Rapid Risk Assessment: Resurgence of reported cases of COVID 19 in the EU/EEA, the UK and EU candidate and potential candidate countries European Centre for Disease Prevention and Control (2020).https://www.ecdc.europa.eu/sites/default/files/documents/RRA-Resurgence-of-reported-cases-of-COVID-19-in-the-EU-EEA.pdf

[18] Farahani SS, Raman V, Murray RM (2015). Robust model predictive control for signal temporal logic synthesis. IFAC Pap. Online.

[19] Feng Z (2014). Applications of Epidemiological Models to Public Health Policymaking: The Role of Heterogeneity in Model Predictions.

[20] Ferguson N.M., et al.: Report 9—impact of non-pharmaceutical interventions (NPIs) to reduce COVID-19 mortality and healthcare demand. Imperial College London (2020).https://www.imperial.ac.uk/mrc-global-infectious-disease-analysis/covid-19/report-9-impact-of-npis-on-covid-19 10.1007/s11538-020-00726-x PMC7140590 32270376

[21] Flaxman S, Mishra S, Gandy A (2020). Estimating the effects of non-pharmaceutical interventions on COVID-19 in Europe. Nature.

[22] Giordano G, Blanchini F, Bruno R, Colaneri P, Di Filippo A, Di Matteo A, Colaneri M (2020). Modelling the COVID-19 epidemic and implementation of population-wide interventions in Italy. Nat. Med..

[23] Golding, N., Russell, T.W., Abbott, S., Hellewell, J., Pearson, C.A., van Zandvoort, K., Jarvis, C.I., Gibbs, H., Liu, Y., Eggo, R.M. Edmunds, J.W.: Reconstructing the global dynamics of under-ascertained COVID-19 cases and infections. medRxiv (2020). 10.1101/2020.07.07.20148460 10.1186/s12916-020-01790-9 PMC7577796 33087179

[24] Hale, T., Angrist, N., Petherick, A., Phillips, T., Webster, S.: Variation in government responses to COVID-19. Blavatnik School of Government Working paper, BSG-WP-2020/032 (2020). https://www.bsg.ox.ac.uk/research/research-projects/coronavirus-government-response-tracker

[25] Heemels WPMH, Daafouz J, Millerioux G (2010). Observer-based control of discrete-time LPV systems with uncertain parameters. IEEE Trans. Autom. Control.

[26] Helmholtz-Initiative ‘Systemische Epidemiologische Analyse der Covid-19-Epidemie’, Stellungnahme der Helmholtz-Initiative ‘Systemische Epidemiologische Analyse der COVID-19-Epidemie’ (2020)

[27] Hernandez-Vargas EA (2019). Modeling and Control of Infectious Diseases in the Host: With MATLAB and R.

[28] Ibeas, A., de la Sen, M., Alonso-Quesada, S., Zamani, I., Shafiee, M.: Observer design for seir discrete-time epidemic models. In: 2014 13th International Conference on Control Automation Robotics and Vision (ICARCV), pp. 1321–1326. IEEE, New York (2014)

[29] Iggidr A, Souza MO (2019). State estimators for some epidemiological systems. J. Math. Biol..

[30] Isidori A (1999). Nonlinear Control Systems.

[31] Khajavirad A, Sahinidis NV (2018). A hybrid LP/NLP paradigm for global optimization relaxations. Math. Program. Comput..

[32] Köhler, J., Schwenkel, L., Koch, A., Berberich, J., Pauli, P., Allgöwer, F.: Robust and optimal predictive control of the COVID-19 outbreak. Preprint (2020). arXiv:2005.03580 10.1016/j.arcontrol.2020.11.002 PMC7757387 33362428

[33] Kronqvist J, Bernal DE, Lundell A, Grossmann IE (2019). A review and comparison of solvers for convex MINLP. Optim. Eng..

[34] KSH: Hungarian Central Statistical Office. http://www.ksh.hu/?lang=en

[35] Lauer SA, Grantz KH, Bi Q, Jones FK, Zheng Q, Meredith HR, Lessler J (2020). The incubation period of Coronavirus disease 2019 (COVID-19) from publicly reported confirmed cases: estimation and application. Anna. Intern. Med..

[36] Löfberg, J.: YALMIP: a toolbox for modeling and optimization in MATLAB. In: Proceedings of the CACSD Conference (2004)

[37] Morato, M.M., Bastos, S.B., Cajueiro, D.O., & Normey-Rico, J.E.: An optimal predictive control strategy for COVID-19 (SARS-CoV-2) social distancing policies in Brazil. Preprint arXiv:2005.10797 [q-bio.PE] (2020) 10.1016/j.arcontrol.2020.07.001 PMC7388786 32837241

[38] Millerioux G, Rosier L, Bloch G, Daafouz J (2004). Bounded state reconstruction error for LPV systems with estimated parameters. IEEE Trans. Autom. Control.

[39] Moghadas SM (2020). Projecting hospital utilization during the COVID-19 outbreaks in the United States. Proc. Natl. Acad. Sci. USA.

[40] Moss, R., et al.: Modelling the impact of COVID-19 in Australia to inform transmission reducing measures and health system preparedness. medR$$\chi $$iv (2020). 10.1101/2020.04.07.20056184 10.3201/eid2612.202530 PMC7706956 32985971

[41] Muqbel K, Vas G, Röst G (2020). Periodic orbits and global stability for a discontinuous SIR model with delayed control. Qual. Theory Dyn. Syst..

[42] Nowzari C, Preciado VM, Pappas GJ (2016). Analysis and control of epidemics: a survey of spreading processes on complex networks. IEEE Control Syst. Mag..

[43] Pugliese, A., Sottile, S.: Inferring the COVID-19 infection curve in Italy. Preprint (2020). arXiv:2004.09404

[44] Riobello, R.N.: On some new mathematical models of infectious diseases: analysis, equilibrium, positivity and vaccination controls. Ph.D. Thesis, University of Basque Country, Spain (2015)

[45] Röst G, Bartha F, Bogya N, Boldog P, Dénes A, Ferenci T, Horváth JK, Juhász A, Nagy C, Tekeli T, Vizi Z, Oroszi B (2020). Early phase of the COVID-19 outbreak in Hungary and post-lockdown scenarios. Viruses.

[46] Sebastiani G, Massa M, Riboli E (2020). COVID-19 epidemic in Italy: evolution, projections and impact of government measures. Eur. J. Epidemiol..

[47] Sélley F, Besenyei Á, Kiss IZ, Simon PL (2015). Dynamic control of modern, network-based epidemic models. SIAM J. Appl. Dyn. Syst..

[48] Sontag ED (1998). Mathematical Control Theory: Deterministic and Finite Dimensional Systems.

[49] Stewart G, Heusden K, Dumont GA (2020). How control theory can help us control COVID-19. IEEE Spectr..

[50] Stoecklin SB (2020). First cases of Coronavirus disease 2019 (COVID-19) in France: Surveillance, investigations and control measures. Euro Surveill..

[51] Ullah S, Khan M (2020). Modeling the impact of non-pharmaceutical interventions on the dynamics of novel Coronavirus with optimal control analysis with a case study. Chaos Solitons and Fractals.

[52] Wang H, Wang Z, Dong Y (2020). Phase-adjusted estimation of the number of Coronavirus disease 2019 cases in Wuhan. China. Cell Discov..

[53] Wang Z, Röst G, Moghadas SM (2019). Delay in booster schedule as a control parameter in vaccination dynamics. J. Math. Biol..

[54] Watkins NJ, Nowzari C, Pappas GJ (2020). Robust economic model predictive control of continuous-time epidemic processes. IEEE Trans. Autom. Control.

[55] Weitz, J.S.: COVID-19 epidemic risk assessment for Georgia. Github (2020). https://github.com/jsweitz/covid-19-ga-summer-2020

[56] Wiersinga WJ, Rhodes A, Cheng AC, Peacock SJ (2019). Pathophysiology, transmission, diagnosis, and treatment of Coronavirus disease 2019 (COVID-19): a review. JAMA.

[57] WHO Situation Report-191, Coronavirus disease 2019 (COVID-19) 29 July 2020. https://www.who.int/docs/default-source/coronaviruse/situation-reports/20200729-covid-19-sitrep-191.pdf

[58] Wu J, Tang B, Bragazzi N, Nah K, McCarthy Z (2020). Quantifying the role of social distancing, personal protection and case detection in mitigating COVID-19 outbreak in Ontario, Canada. J. Math. Ind..

[59] Zou L, Ruan F, Huang M (2020). SARS-CoV-2 viral load in upper respiratory specimens of infected patients. N. Engl. J. Med..

